# Barriers against the use of an optimal dose of metformin among patients with T2DM in Thi-Qar province, Iraq

**DOI:** 10.25122/jml-2021-0259

**Published:** 2022-04

**Authors:** Dheyaa Al-Waeli, Adel Mohammed, Imad Tahir, Ali Al-Saeedi, Khdair Razzaq, Ali Abodhurais

**Affiliations:** 1. Department of Medicine, College of Medicine, University of Thi-Qar, Nasiriyah, Thi-Qar, Iraq; 2. Thi-Qar Specialized Diabetes, Endocrine and Metabolism Center (TDEMC), Thi-Qar Health Directorate, Nasiriyah, Thi-Qar, Iraq; 3. Department of Internal Medicine, Al-Hussein Teaching Hospital, Thi-Qar Health Directorate, Nasiriyah, Thi-Qar, Iraq

**Keywords:** Metformin, Diabetes mellitus, Adherence, Barriers

## Abstract

Diabetes mellitus is a disease with a high burden and prevalence and serious complications. Glycemic control is vital in delaying or preventing complications. Although many people do not take optimal doses, metformin is a cornerstone in managing type 2 diabetes mellitus (T2DM) in all guidelines. This study determined the barriers interfering with optimal metformin dosage. A cross-sectional study was conducted in Thi-Qar Specialized, Diabetes, Endocrine and Metabolism Center (TDEMC) at Thi-Qar, southern Iraq, from January 2019 to January 2020. 475 patients (274 females and 201 males) were included, and examination and lab investigations were performed. Only 22 (4.6%) patients took the optimal dose with no differences between gender. Of those who took metformin, 255 (74%) took it as a regular pill, 79 (23%) as a combined form with sulfonylureas (SUs), while only 10 (0.3%) took combined pills with Dipeptidyl Peptidase-4 inhibitors (DPP4i). 188 patients (65%) took metformin with meals, 84 (29%) before meals, and 19 (6%) after meals. Ignorance caused poor adherence to optimal dose in 165 patients (38.6%), neglect in 75 (17.6%), the cost in 5 (1.2%), 11 patients (2.6%) thought they did not need metformin, 37(8.7%) and 12 (2.8%) blame side effects and shortage of supply from public health care clinics (PHCC) as a cause, respectively. The rest of the patients had more than one cause. The most common side effects were abdominal pain and bloating, 5.9% and 3.8%, respectively. Other side effects were diarrhea in 0.2%, and 7.8% of patients developed more than one side effect. Ignorance and neglect were major obstacles, so educating doctors and patients and supplying the patient with optimal doses through PHCC may overcome the problem.

## Introduction

Type 2 diabetes mellitus (T2DM) is the most common type of diabetes worldwide; it occurs due to insulin resistance and gradual loss of β-cells functions of the pancreas [1]. According to the International Diabetes Federation (IDF), about 463 million people (8.3% of the adult population) were affected by diabetes in 2019, with the highest prevalence rate in the Middle East and North Africa (MENA) region (12.2%). It is expected that the prevalence of diabetes mellitus (DM) will increase in 2045 to affect about 700 million people worldwide [2]. In Iraq, the prevalence of DM in adult people was 19.7%, as concluded by a study performed in a southern city [3], while IDF reported 1.4 million Iraqis have T2DM with a prevalence of 8.5–13.9% [4]. Diabetes represents a significant health problem in Iraq due to its increasing prevalence and incidence, the impact on people's economic and health status, and the chronicity of the disease [5]. Strict glycemic control plays a very important role in reducing both mortality and morbidity of diabetes, with the HbA1c target of 7% being indicated by many associations as a guide for good glycemic control [6]. Unfortunately, despite the heavy burden of diabetes on quality of life, most patients (about 86%) in the south of Iraq did not achieve targeted glycemic control [7].

All guidelines recommend metformin with lifestyle modification or in combination with other drugs as the preferred initial treatment in the management of diabetes unless it is contraindicated [6]. It is affordable, safe, and effective, and it has a useful effect on HbAIc. Furthermore, it has a beneficial effect on weight reduction and may be associated with less cardiovascular morbidity and mortality [8, 9]. The best dose for these beneficial effects is 2000 mg daily [10]. Unfortunately, many of our patients showed poor adherence to metformin and did not take this optimal dose. For this reason, we performed this study at the Thi-Qar Specialized Diabetes, Endocrine, and Metabolism Center (TDEMC) in Thi-Qar province, south of Iraq (which is a tertiary center that receives patients from all over Thi-Qar province) to evaluate patient barriers against taking the optimal metformin dose.

## Material and methods

A cross-sectional study was performed at TDEMC from January 2019 to January 2020 to collect information from patients who visited our center through organized interviews constructed on a pre-evaluated questionnaire. It included 475 (274 females and 201 males) patients with type 2 diabetes mellitus (T2DM), selected randomly from people who attended the center all over Thi-Qar province. Data such as socio-demographics, duration of diabetes, co-morbidities, dosage, formula, time of metformin intake, side effects, and most common barriers against compliance with optimal metformin dose were collected. Full clinical examination was performed, including vital signs and body mass index (BMI) measurements, followed by biochemical investigations like HbA1c, random or fasting blood sugar, renal function, and lipid profile. 

### Statistical analysis

Excel sheet and SPSS Statistics 25.0 were used for data analysis. Descriptive statistics, frequencies, percentages, associations, and tests of significance (ANOVA) were used to analyze quantitative-continuous variables. Means and standard deviations were used to present continuous variables. Chi-square test was used for qualitative variables. A P-value<0.05 was considered statistically significant.

## Results

**Figure 1.**
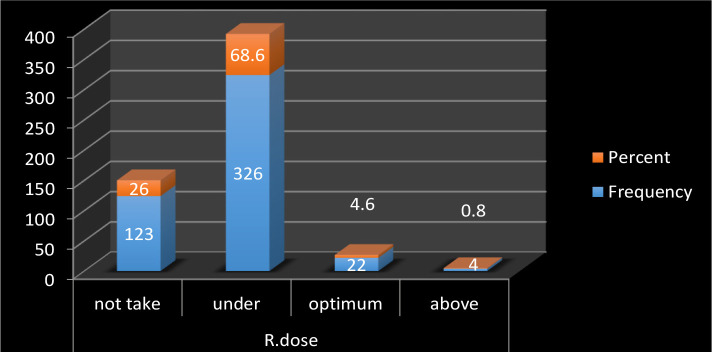
Distribution of the sample according to metformin dose adherence. R dose – recommended dose -2000 mg/day.

The mean age of our cohort was 56.2±9.9 years, the mean duration of diabetes was 10±6.6 years, and the mean body mass index was 30.6±6 (Table 1). The present study showed that only 22 (4.6%) patients took an optimal dose of metformin (2000 mg), 326 (68.6%) patients took a suboptimal dose, 4 (0.8%) take more than 2g, while 123 (26%) patients were not taking metformin at all (Figure 1). There was no statistically significant difference between males and females regarding dose optimality (p=0.2).

**Table 1.** Descriptive statistics of studied population.

	**Min.**	**Max.**	**Mean**	**S. D**	**ANOVA**	**p**
**Age by years**	23	82.	56.247	9.99	1.398	0.172
Duration of DM*	1	39	10.984	6.64	.389	.960
**BMI (kg/m^2^)**	10	51	30.769	6.60	2.291	.010
Duration**	1	34	5.763	4.16	1.405	.170
**Metformin dose (mg)**	500	2850	729.789	600.73	400.538	.0001
**HbA1c %**	6.01	18.10	9.893	2.648	.156	.926
**Cholesterol (mg/dL)**	84	300	188.065	41.68	2.618	.055
**Tg (mg/dL)**	80	952	180.710	129.36	.419	.740
**FBS(mg/dL)**	50	504	196.730	79.87	.954	.415
**RBS(mg/dL)**	60	600	260.732	101.77	.347	.791
**BU(mg/dL)**	8	60	34.056	8.96	1.627	.189

* – Duration of DM in months; ** – Duration of registration in the center.

Most patients (255 patients) took metformin in the form of regular pills, with 11 patients (4.3%) taking an optimal dose (p=0.01), 79 patients took metformin as combined pills with sulfonylureas (SUs) (10 of them taking optimal dose), and 10 patients took metformin as combined pills with Dipeptidyl Peptidase-4 inhibitors (DPP4i) (none having optimal dose) as shown in Table 2. There was a significant statistical difference between patients taking an optimal dose (21 [6.1%]) and those taking a suboptimal dose (319 [92%]) regarding all types of formulations (p=0.01).

**Table 2.** State of metformin intake.

**State of metformin intake**	**Dose**			**Total**	**X^2^**
	**Under**	**Optimal**	**>2000**		**P-value**
**Only Metformin**	242	11	2	255	14.560 0.01
	94.9%	4.3%	0.8%	100.0%	
**Combined-SU**	67	10	2	79	
	84.8%	12.7%	2.5%	100.0%	
**Combined-DPP4**	10	0	0	10	
	100.0%	0.0%	0.0%	100.0%	
**Total**	319	21	4	344*	
	92.7%	6.1%	1.2%	100.0%	

* – 8 missed cases (the question was not answered properly) and 123 patients not taking metformin at all.

As shown in Table 3, eighty-four (28.8%) patients took metformin before meals (only 6 of them taking optimal dose), 19 patients took it after meals (only one of them taking an optimal dose), while the majority of our patients (188) took metformin with the meals (only 13 of them taking an optimal dose) with a significant statistical difference (p=0.001). There was a significant statistical difference between patients taking an optimal dose (20 [6.9%]) and undertreated patients (267 [91.7%]) at different times of taking metformin (p=0.001).

**Table 3.** Time of taking metformin according to the dose.

**Time of taking metformin**		Time of taking metformin * dose				**X^2^**
		**Dose**			**Total**	**P-value**
		**Under**	**Optimal**	**>2000**		
**Before meal**	Count	78	6	0	84	26.99 0.001
	% within	92.9%	7.1%	0.0%	100.0%	
**Within meal**	Count	171	13	4	188	
	% within	91.0%	6.9%	2.1%	100.0%	
**After meal**	Count	18	1	0	19	
	% within	94.7%	5.3%	0.0%	100.0%	
**Total**	Count	267	20	4	291*	
	% within	91.7%	6.9%	1.4%	100.0%	

* – 61 cases not accurately answering the question and 123 patients not taking metformin.

**Figure 2.**
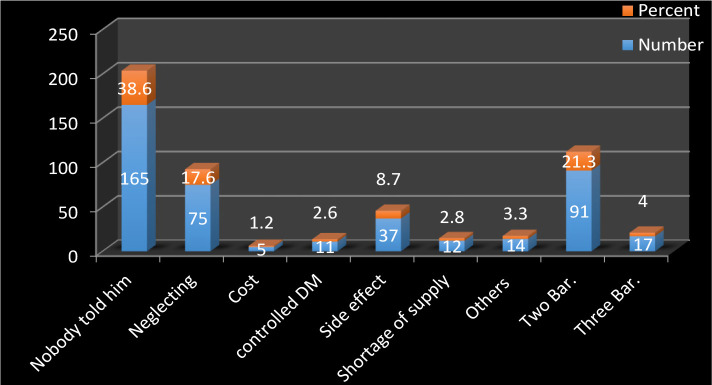
Main barriers against taking an optimal dose of metformin (22 patients not answering the question appropriately, 22 patients take optimal doses, and 4 patients take more than 2000 mg).

The most common cause for not taking the optimal dose of metformin (38.6% of patients) was poor patient knowledge, followed by patients with 2 barriers (21.1%) and neglect (17.6%). Other barriers represent a small percentage of patients. There was a significant difference (p=0.001) in the distribution of barriers among those who take sub-optimal dosage and those who do not take metformin (Figure 2, Table 4).

**Table 4.** Barriers against the use of an optimal dose of metformin.

**Barriers**		**Dose**			**FE**
		**Not take**	**Under**	**Total**	**P-value**
**Nobody told him**		11	152	163	39.872 0.001
		6.7%	93.3%	100.0%	
**Neglecting**		30	44	74	
		40.0%	60.0%	100.0%	
**Cost**		1	3	4	
		25.0%	75.0%	100.0%	
**Side effect**		18	19	37	
		48.6%	51.4%	100.0%	
**Controlled DM**		1	10	11	
		9.0%	91.0%	100.0%	
**Shortage of supply**		9	3	12	
		75.0%	25.0%	100.0%	
**Others**		2	0	2	
		100.0%	0.0%	100.0%	
**Two Barriers**		30	60	90	
		33.3%	66.7%	100.0%	
**Three Barriers**		5	12	17	
		29.4%	70.6%	100.0%	
**Total**	Count	107	303	410*	
	**Percent**	26.1	73.9%	100.0%	

* – 39 cases not accurately answering the question or inappropriate history taking. FE – Fisher's exact test.

**Figure 3.**
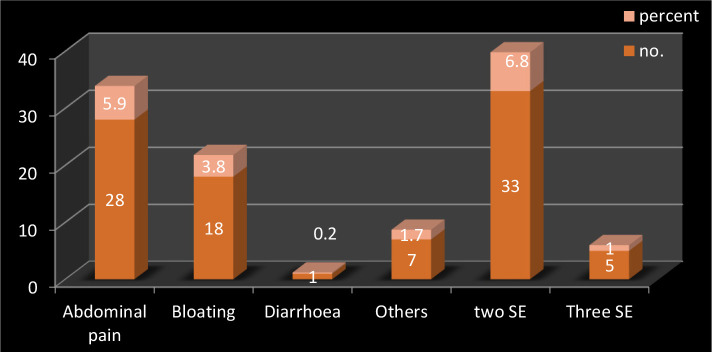
Common side effects of metformin. Two SE (two side effects); Three SE (three side effects).

Abdominal pain was the most common and statistically significant side effect (p=0.007), which occurred in 28 patients, bloating in 18 patients, one patient developed diarrhea, seven patients developed other side effects, 33 patients showed 2 of the above side effects, and 5 patients developed 3 side effects at the same time (Figure 3).

134 patients had hypertension (3% of them took an optimal dose of metformin), 17 patients had ischemic heart disease (17.6% of them taking the optimal dose, 2 patients had heart failure (one on optimal dose), one patient had renal failure (on full dose).

Thirty-five patients had peripheral neuropathy (only one of them (2.9%) on optimal dose), 4 patients with erectile dysfunction (one of them on optimal dose), 5 patients with diabetic foot (none on optimal dose), 115 patients with 2 or more co-morbidities (5 of them took an optimal dose of metformin) (Table 5). There was a significant statistical association between the suboptimal dose of metformin and the presence of co-morbidities in diabetic patients (p=0.007).

**Table 5.** Relationship of comorbid conditions with metformin dose

**Co-morbidities**		**Dose**				**Total**	**FE**
		**None**	**Sub**	**Optimal**	**Over**		**P-value**
**HTN**	Count	31	99	4	0	134	203.543 0.023
	% within	23.1%	73.9%	3.0%	0.0%	100%	
**IHD**	Count	2	12	3	0	17	
	% within	11.8%	70.6%	17.6%	0.0%	100%	
**HF**	Count	0	1	1	0	2	
	% within	0.0%	50.0%	50.0%	0.0%	100.0%	
**RF**	Count	0	1	0	0	1	
	% within	0.0%	100.0%	0.0%	0.0%	100.0%	
**PNP**	Count	9	25	1	0	35	
	% within	25.7%	71.4%	2.9%	0.0%	100.0%	
**ED**	Count	2	1	1	0	4	
	% within	50.0%	25.0%	25.0%	0.0%	100.0%	
**DF**	Count	3	2	0	0	5	
	% within	60.0%	40.0%	0.0%	0.0%	100.0%	
**Two**	Count	22	53	5	1	81	
	% within	27.2%	65.4%	6.3%	1.1%	100.0%	
**Three**	Count	10	24	0	0	34	
	% within	29.4%	70.6%	0.0%	0.0%	100.0%	
Total	Count	79	218	15	1	313	
	% within	25.2%	69.7%	4.8%	0.3%	100.0%	

FE – Fisher's exact test.

## Discussion

As identified by the World Health Organization (WHO) and many studies, there were many factors affecting the adherence of the patient to their medications, including age, gender, motivation, ethnicity, socioeconomic status (cost of drugs, level of education), related circumstances (other chronic illnesses, duration of diabetes, presence or absence of complications), health care system (health care public clinics, pharmacies), treatment factors (side effects, polypharmacy and bad past personal or family history with the drugs) [11, 12].

In this study, the most common cause of poor adherence to optimal metformin dose was ignorance with regards to this issue which might be due to poor communication with their doctors or even unawareness of the doctors or health care practitioners themselves about the importance of this practice (because many patients told us that nobody advised them to take the optimal dose). Low educational level was a cause of poor adherence to treatment in many other studies [12, 13]. In this study, only 1.2% of poor adherent patients considered cost as a cause of insufficient dose of metformin, while in other studies, the effect of cost reached 7% [14] and even 51.3% [15]. The low effect of cost in our study may be explained by the fact that there are many generic types of metformin in the markets that most patients find affordable. In addition, public health clinics supply the patients with monthly metformin supplements; although these are not enough, they help the patients provide part of their treatments. Also, these two studies included other antidiabetic medications rather than metformin which may explain the effect of cost in these studies because these drugs are relatively more expensive than metformin. 

In a French study, the treatment adherence was better than in our study. The most common causes in poorly adherent patients were socio-demographic influence, disease and treatment associated factors, and health care factors that differ from our study due to different population criteria [16]. Our study agreed with a Pakistanis study that concluded that shortage of knowledge, economic resources, and poor compliance were the leading causes of poor adherence to treatment [13].

Most of our patients took metformin in regular forms rather than in combination pills with SUs or DPP4i, most likely due to the cost-effectiveness and availability of regular metformin (rather than the combination formula) in the public health sector. The majority of those taking combination formula did not take the optimal dose because they tried to decrease the cost of medication, considering that these combinations contain two drugs and there is no need to take more than once. Therefore, ignorance and cost are the main barriers in those patients, as noticed in many studies [13, 16]. 

Although most of our patients took metformin with meals, the majority did not take an optimal dose, which means that the time of taking the drug has no important role in adherence because the most important cause was unawareness of the patients regarding dose optimality rather than drug side effects.

The most common side effect of metformin in our study was gastro-intestinal tract (GIT) side effects which developed in about 20% of patients, coinciding with previous studies [17]. 

The increased prevalence of co-morbidities in those patients taking suboptimal doses of metformin may be explained by a beneficial effect of this drug in diabetic patients, an effect confirmed by previous studies [18, 19].

## Conclusion

Unawareness of the patients regarding the beneficial and optimal dose of metformin, side effects, and the cost were the most common causes of poor compliance of patients. Education of both patients and doctors regarding the importance of this issue, instructions about the best time of taking metformin in relation to meals, and providing the patient with optimal, affordable (and, if possible, with the extended-release formula of 1000 mg to decrease GIT side effects and ensure compliance) doses through public health care clinics may help overcome this problem.

## Acknowledgments 

### Conflict of interest

The authors declare no conflict of interest.

### Ethical approval

The study was approved by the Ethical Committee of Thi-Qar Specialized Diabetes, Endocrine, and Metabolism Center (67/36/21).

### Consent to participate

All participants received written informed consent before participating in the study.

### Personal thanks

All authors are grateful to the laboratory personnel of TDEMC for their assistance and encouragement.

### Authorship

DA-W contributed to conceptualizing, methodology and writing. AM contributed to methodology and editing. IT contributed to data collection, curation, and analysis. AA-S contributed to data collection and analysis. KR contributed to data collection, curation, and analysis. AKA contributed to methodology, editing and writing.
